# Tolerance of a standard intact protein formula versus a partially hydrolyzed formula in healthy, term infants

**DOI:** 10.1186/1475-2891-8-27

**Published:** 2009-06-19

**Authors:** Carol Lynn Berseth, Susan Hazels Mitmesser, Ekhard E Ziegler, John D Marunycz, Jon Vanderhoof

**Affiliations:** 1 Mead Johnson Nutrition, 2400 W. Lloyd Expressway, Evansville, Indiana, 47721, USA; 2 Department of Pediatrics, University of Iowa Children's Hospital, Iowa City, Iowa 52242, USA

## Abstract

**Background:**

Parents who perceive common infant behaviors as formula intolerance-related often switch formulas without consulting a health professional. Up to one-half of formula-fed infants experience a formula change during the first six months of life.

**Methods:**

The objective of this study was to assess discontinuance due to study physician-assessed formula intolerance in healthy, term infants. Infants (335) were randomized to receive either a standard intact cow milk protein formula (INTACT) or a partially hydrolyzed cow milk protein formula (PH) in a 60 day non-inferiority trial. Discontinuance due to study physician-assessed formula intolerance was the primary outcome. Secondary outcomes included number of infants who discontinued for any reason, including parent-assessed.

**Results:**

Formula intolerance between groups (INTACT, 12.3% vs. PH, 13.7%) was similar for infants who completed the study or discontinued due to study physician-assessed formula intolerance. Overall study discontinuance based on parent- vs. study physician-assessed intolerance for all infants (14.4 vs.11.1%) was significantly different (P = 0.001).

**Conclusion:**

This study demonstrated no difference in infant tolerance of intact vs. partially hydrolyzed cow milk protein formulas for healthy, term infants over a 60-day feeding trial, suggesting nonstandard partially hydrolyzed formulas are not necessary as a first-choice for healthy infants. Parents frequently perceived infant behavior as formula intolerance, paralleling previous reports of unnecessary formula changes.

**Trial Registration:**

clinicaltrials.gov: NCT00666120

## Background

Breastfeeding is the ideal source of nutrition for infants, but standard cow milk-based formulas (CMBF) are an appropriate source of nutrition for most infants who are not breastfed [1]. Some nonstandard formulas, such as soy-based or partially or extensively hydrolyzed protein cow milk-based formulas (CMBF), may be indicated for select groups of infants [2-4]. Partially hydrolyzed formulas are marketed as easier to digest although no evidence supports the physical need for these formulas in healthy infants. In addition, healthy infants who receive a CMBF with intact protein are often unnecessarily switched to a nonstandard formula or alternate CMBF [5-7]. Although published estimates of formula intolerance range from 2 to 15% [8,9], one-third to one-half of infants, however, undergo formula change during the first 6 months of life [5,6], implying unnecessary switching or use of nonstandard formulas in healthy term infants. Rather than a pediatrician, parents or caregivers made 47% to 96% of decisions to discontinue a formula based most often on misinterpretation of common infantile behavior patterns as formula intolerance-related [5,6].

The primary objective of this non-inferiority study was to compare tolerance of a standard, full-lactose non-hydrolyzed cow milk protein formula and a 70% lactose non-standard partially hydrolyzed whey protein formula over a 60 day period in healthy, term infants. Non-inferiority or equivalence studies are designed to determine if the efficacy of new intervention matches that of a previously used or accepted routine intervention. In this study, the primary outcome was formula intolerance, indirectly reflected by the number of infants who discontinued study formula due to study physician-determined intolerance, to test the hypothesis that there is no advantage in choosing a partially hydrolyzed protein CMBF as a first-choice formula for healthy infants. Secondary outcomes compared between formula groups included: discontinuation of study formula for any reason, including parental assessment of formula intolerance and infant temperament using the validated Infant Characteristic Questionnaire [10].

## Materials and methods

### Study design

This was a multicenter, double-blind, randomized, controlled, parallel, prospective, non-inferiority trial. A total of 335 healthy, term infants were randomized to receive one of two study formulas: a full lactose, intact cow milk protein (60:40 whey:casein) formula supplemented with docosahexaenoic acid (DHA) and arachidonic acid (ARA) (INTACT; Enfamil^® ^LIPIL^®^, Mead Johnson Nutrition, Evansville, IN) or a 70% lactose, partially hydrolyzed cow milk protein (100:0 whey:casein) formula supplemented with DHA and ARA (PH; Good Start^® ^Supreme, Nestlé^® ^S.A., Vevey, Switzerland) over a 60 day period. INTACT is a routine formula intended for healthy, term infants without special nutritional needs and is patterned upon the protein profile of human breast milk [11]. PH is also promoted as a routine infant formula and is formulated with partially hydrolyzed, 100% whey proteins. Outpatient visits were conducted at enrollment (randomization to study formula), day 30, and day 60; a telephone interview of the parent or caretaker was conducted on day 45. The study was approved by a central or site-specific Institutional Review Board. Parents or guardians provided written informed consent prior to study participation.

A randomization list for each study site was created by the study sponsor using a computerized random-number generator. Two different codes, known only to the study sponsor, designated each study formula. Sealed envelopes labeled with consecutive numbers contained the code of the study formula that was to be assigned based on the randomization list. Upon entry into the study the next sequential numbered envelope was opened at the study site to provide the code of the study formula to be consumed by the infant. Study formula, packaged by the study sponsor as identifiable only by its code, was then provided to the infant.

### Study population

Eligibility criteria included: term, singleton-birth infants; birth weight of > 2500 g; 4 to 18 days of age; infants had solely received a full-lactose, standard, intact protein CMBF ≥ 96 hours prior to enrollment; and the mother of the infant had no plans to breastfeed during the study. Enrollment took place at 17 research centers and pediatric offices throughout the United States (detailed in Acknowledgements) between June 13, 2005, and February 27, 2006. Exclusion criteria included a history of underlying metabolic or chronic disease or congenital malformation likely to interfere with normal growth and development; known feeding problems; switching formulas more than one time between birth and enrollment; parent-reported known history of cow milk allergy in parent or siblings; and use of commonly-prescribed medications for gas in infants at enrollment.

### Outcomes

The primary outcome was discontinuation of assigned study formula due to physician-assessed feeding intolerance. Specified criteria for a diagnosis of formula intolerance included the presence of one or more of the following: vomiting, fussiness, allergic reaction, hunger, constipation, diarrhea, gas, or spitting up.

Body weight, length, and head circumference were measured at enrollment. Body weight was measured again at days 30 and 60. All other secondary outcomes were parent- or caregiver- reported. Formula intake and tolerance (spit up, crying, fussiness, gas, stool frequency, and stool consistency) were collected using a 24 h recall questionnaire at clinic visits on days 30 and 60 and during a telephone interview at day 45. Number of infants per group who discontinued study formula due to parental assessment or for any reason was collected at day 60. The widely-used, validated Infant Characteristic Questionnaire (ICQ) [10] assessed parental perception of infant difficultness. Fussy, difficult-to-soothe infants with irregular behavior are often regarded as difficult. The 24-question ICQ focuses on four subscales of infant behavior (questions per scale): fussy-difficult (9), adaptable (5), dull (4), and predictable (6) with the most weight given to fussy-difficult. The ICQ subscales address infant difficultness based on fussy behavior; consequently, fussy behavior associated with formula intolerance and potential differences in infant fussiness due to feeding regimen were examined with this instrument. All responses were measured on a 7-point scale (1 = optimal temperament to 7 = difficult temperament) and scores were calculated by summing responses in each subscale. Feeding satisfaction (parental perceptions of infant feeding behavior, feelings of parent/caregiver regarding feeding success, and success as a caregiver) was assessed using a 15-question Formula Feeding Satisfaction Questionnaire (FSQ). Responses were measured on a 7-point scale (1 = strongly agree to 7 = strongly disagree) and totaled (maximum score = 105; some questions were reverse-coded for analysis, thus a higher total score indicated higher formula satisfaction for all items of the questionnaire). The FSQ and ICQ were administered at enrollment and day 60, or if a participant discontinued the study early. Adverse events were monitored throughout the study.

### Statistical analyses

In this non-inferiority study, the sample size was determined based on dropout rates reported in previous clinical studies [12,13] to detect a difference in discontinuation rate due to formula intolerance of 30% versus 15% (α = 0.05, power = 80%). A total of 134 infants per formula group (classified as completed the study through day 60 or discontinued due to formula intolerance) was determined to be sufficient. Two datasets were analyzed: 1) the per protocol dataset (D-I), included only infants who completed the study through day 60 or discontinued due to study physician-determined formula intolerance; 2) secondary dataset (D-II), included all study participants who consumed study formula. This distinction was made in order to determine the primary outcome of discontinuation rate due to study-physician assessment of formula intolerance excluding other reasons for study discontinuance. Consequently, the Cochran-Mantel-Haenszel test stratified by study site was performed on the D-I dataset to compare rates of discontinuation due to study physician-determined formula intolerance and on the D-II dataset to compare rates of discontinuation for all reasons, including parental opinion of formula intolerance. The McNemar test was performed on the D-II dataset to compare study physician vs. parental opinion on whether study discontinuation was due to formula intolerance.

Formula intake and tolerance from the 24 h recall questionnaire were compared between formula groups at day 30, day 45, and day 60. The analysis of variance model for formula intake included study site, gender, formula group, and gender by formula group interaction. Tolerance measures with continuous data included only study site and formula group in the analysis of variance model. Categorical data were analyzed using Fisher's exact test. ICQ subscales were analyzed by repeated measures analysis of variance. Study groups were compared at enrollment and day 60. Comparisons were made within groups from enrollment to day 60. Fisher's exact test was used to compare the proportion of infants in each formula group with serious adverse events during the study using the D-II dataset.

Statistical comparisons were two-tailed and all testing was conducted at α = 0.05. Analyses were performed by using SAS version 9 (Cary, NC).

## Results

**Figure 1:**
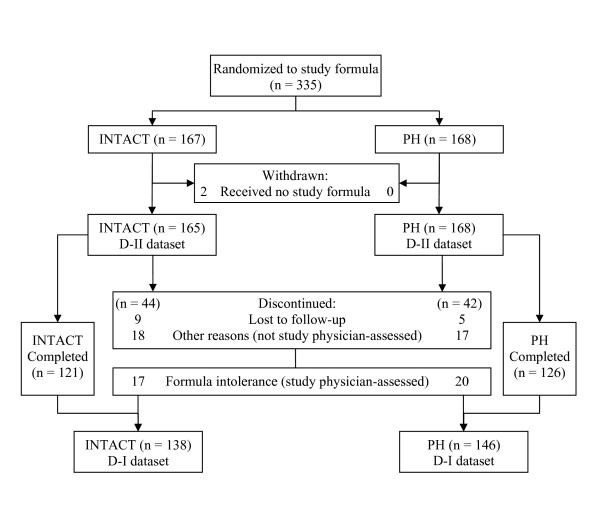
**Flow of study participants**.

A total of 335 infants enrolled at 17 study sites between June 2005 and February 2006 (INTACT, 167; PH, 168); two withdrew prior to receiving any study formula (Fig. 1). The D-I dataset included 284 infants (INTACT, n = 138; PH, n = 146) and the D-II dataset included 333 infants (INTACT, n = 165; PH, n = 168). Ten infants, enrolled in the study and included in analyses, did not meet the protocol inclusion criteria (INTACT, 2; PH, 8). Two of the ten had birth weights less than the required minimum (2500 g). One had a parent/sibling with a history of cow milk allergy. Seven did not meet the minimum 38 week gestational age. Also, one of these seven may have consumed breast milk sometime during the 96 hours prior to enrollment.

**Figure 2:**
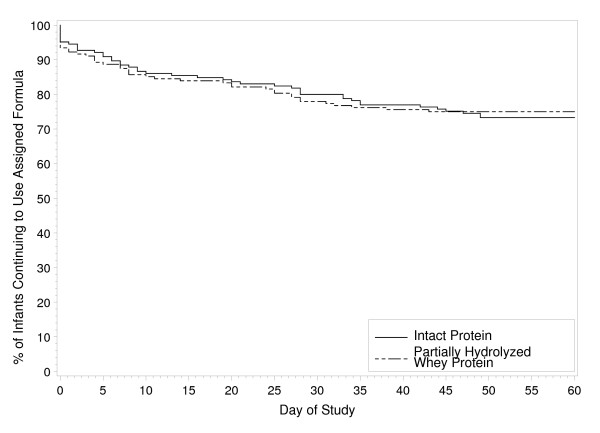
**Percentage of infants that continued to consume INTACT formula (full line) or PH formula (dashed line) from enrollment (day 1 of study) to day 60**.

Infant characteristics are summarized in Table 1. Infants in both formula groups were similar at enrollment for gender, race, and anthropometrics. Study completion rates [INTACT, 121 (73%) of 165; PH, 126 (75%) of 168; *P *= 0.731], the percentage of infants who remained in the study throughout the 60 day feeding period (Fig. 2), and study physician-assessed formula intolerance [INTACT, 17 (12.3%) of 138; PH, 20 (13.7%) of 146; *P *= 0.812, D-I dataset; 95% CI, -9.3% to 6.5%] (Fig. 1) were similar between groups. Consequently, there was no significant difference between groups in discontinuance for the primary outcome of study physician-assessed formula intolerance. Of the 37 infants who discontinued due to physician-assessed formula intolerance, the main symptoms reported were fussiness (n = 11 vs. 15), gas (n = 11 vs. 14), spitting up (n = 4 vs. 8), vomiting (n = 2 vs. 4), and constipation (n = 2 vs. 3) for INTACT vs. PH, respectively. However, overall study discontinuance based on study physician-assessed versus parent-assessed formula intolerance [37 (11.1%) of 333 vs. 48 (14.4%) of 333] for all infants was significantly different (*P *= 0.001, D-II dataset). That is, more parents deemed their infants to be feeding intolerant than did their physicians.

**Table 1:** Infant characteristics at enrollment

	INTACT	PH
Weight, g*	3571.4 ± 51.15	3533.5 ± 52.17
Length, cm*	51.4 ± 0.23	51.1 ± 0.24
Head circumference, cm*	35.8 ± 0.15	35.7 ± 0.15
Gender, *n*		
Male	94	91
Female	71	77
Race, *n*		
White	115	127
Black	38	27
Other	12	14

* Mean ± standard error (SE)

No significant differences in weight were observed between groups at days 30 or 60 (data not shown). Parental 24 h recall of infant formula intake and tolerance measures at days 30, 45 and 60 were similar (data not shown) with the exception of stool frequency at day 60 (1.9/day, INTACT vs. 2.2/day, PH; *P *= 0.022, D-I and D-II datasets). Low incidence of constipation or diarrhea was similar for both feeding groups (0 – 6% infants affected at any time). Means for ICQ infant temperament subscales (D-I and D-II datasets) were similar between groups at enrollment and study end (day 60 or day of study discontinuation; Table 2). In this healthy infant population, fussy-difficult ICQ scores, in particular, were similar and below mid-range within study groups from enrollment to study end. However, INTACT adaptability scores at study end (ex: reactions to new people, disruptions, bath-time) were significantly lower (better) than at enrollment. INTACT and PH dullness scores at study end (ex: smiling, excitement in play, activity) were also significantly lower than at enrollment. Because a lower ICQ score is closer to optimal behavior it is not surprising that scores in these areas improved with infant age by the end of the feeding trial. Means (± SE) for parental FSQ total scores (maximum possible total score = 105) were similar between groups at enrollment (INTACT, 95.8 ± 1.3; PH, 97.1 ± 1.3) and study end (INTACT, 91.9 ± 1.3; PH, 93.2 ± 1.3). Parents were equally and highly-satisfied with fully intact CMBF formulas at enrollment, (i.e., before study formulas were administered) and with both INTACT and PH by study end.

**Table 2:** Parental perception of infant temperament :  Assessment using the Infant Characteristics Questionnaire (ICQ)* at enrollment and study end^†^

**ICQ subscale**^§^	**enrollment**^‡^		**study end**^‡^	
	
	**INTACT**	**PH**	**INTACT**	**PH**
Fussy-difficult	25.6 ± 0.79	24.7 ± 0.80	25.5 ± 0.81	25.8 ± 0.82
Adaptable	11.8 ± 0.50	11.6 ± 0.51	10.4 ± 0.51^a^	10.9 ± 0.52
Dull	14.8 ± 0.45	15.3 ± 0.46	9.8 ± 0.47^b^	10.2 ± 0.47^b^
Predictable	16.7 ± 0.54	16.6 ± 0.54	16.0 ± 0.55	16.4 ± 0.56

* A lower ICQ score is closer to optimal behavior

^† ^Study end: day 60 (study completion) or early study discontinuation at any time point

^‡ ^Comparisons at time-points between study groups for ICQ subscale were not statistically significant, *P *> 0.05.

^§^Least square means ± SE

^a ^score at enrollment vs. day 60, *P *= 0.001

^b ^score at enrollment vs. day 60, *P *< 0.001

There were no differences in frequency of serious adverse events between formula groups (INTACT, 5/165; PH, 5/168). Serious adverse events for the INTACT group included: cervical lymph adenitis abscess secondary to *Staphylococcus aureus *infection, seizure, respiratory syncytial virus with bronchiolitis, pyloric stenosis, and an apparent life-threatening event (n = 1 per event). Serious adverse events for the PH group included: respiratory syncytial virus, sudden infant death syndrome, moderate gastroesophageal reflux, jaundice and sepsis, and pyloric stenosis (n = 1 per event). All events were judged as unrelated to study formula intake.

## Discussion

The present non-inferiority study demonstrated no significant differences in study discontinuance between infants in the INTACT or PH group based on study physician-assessed formula intolerance over a 60-day feeding trial. Thus, this study indicated that formula tolerance was similar in healthy term infants who received an intact cow milk protein formula or a partially hydrolyzed cow milk protein formula. Using formula discontinuation as an indirect marker of intolerance, this study demonstrated healthy, term infants gained no advantage by receiving a CMBF with partially hydrolyzed whey protein instead of a standard, intact protein CMBF patterned closely on the protein profile of human milk. Other than a difference in stool frequency, groups were similar for infant formula intake, weight, and tolerance (hours of crying, fussiness, gas, stool consistency, and incidence of diarrhea and constipation). Parent-reported FSQ (feeding satisfaction) and all four ICQ temperament subscales were also similar between groups at all measured time-points, indicating comparable formula tolerance for both INTACT and PH groups.

A significant difference in study physician- versus parent-assessed opinion of formula intolerance was noted for all infants randomized to either formula. The results are consistent with reports that parents often discontinue use of a particular formula for reasons other than pediatrician-assessed symptoms associated with formula intolerance. The statistical methods used in this study, including the large number of participants and multi-center design, were adequate to detect this small but significant difference in formula tolerance assessment. Results of this study were similar to previous reports of infant feeding tolerance and formula changes in early infancy [5-7]. In one survey, formulas were changed in over 30% of infants with parent-reported feeding-related issues such as colic, excessive crying, or belief that an infant had a cow milk allergy [7]. In another, parental decision to discontinue a particular formula, rather than a physician or other health care provider, occurred in 47% of infants [6]. Pediatricians were involved in only 4% of decisions to switch formulas in another study where 47% of infants underwent discontinuation of at least one formula within the first 6 months of life [5].

Normal feeding and adaptation between infant and caregiver during the first few weeks of life includes common episodes of regurgitation, crying, fussiness, and colic regardless of feeding regimen [14-17]. Daily regurgitation is reported in up to one-half of infants [14,15]. In a study of infant crying, colic, spit-ups, and feeding difficulties, 35% percent of mothers reported moderate or severe problems in the early postnatal period [16]. Such behaviors in early infancy may be erroneously parent-labeled as formula intolerance and drive the parent to discontinue an infant formula. Other non-behavioral or non-medical factors may also impact parental decisions to change formulas: family or friends' recommendations, parent returning to work, formula cost, or media influence.

Manufacturers of partially hydrolyzed formulas often advertise products as easier to digest. For example, infants fed a whey protein hydrolysate formula had faster gastric emptying compared with infants fed traditional whey-predominant or casein-predominant formulas [18]; however, faster gastric emptying may not equal easier digestion. Kinetics play a major role in dietary nitrogen utilization and slower digestion of milk protein fractions may induce better postprandial nitrogen utilization [19]. In the present study, infants eligible at enrollment solely received a standard, full-lactose, intact protein CMBF for at least 96 hours prior to randomization to INTACT or PH groups. Parental ICQ temperament scores and total feeding satisfaction (FSQ scores) were similar for both groups at enrollment – before an assessment of INTACT vs. PH was undertaken – and at day 60. Fussy-difficult scores in this healthy population did not change from enrollment to study end (day 60 or day of study discontinuation) in either study group. Barring subsequent indications of fussiness or formula intolerance, results from our study suggest that within a normal, healthy infant population there is no need to use a partially hydrolyzed formula instead of a standard CMBF. Given the results from this study and the increased cost of nonstandard formulas [4], suggesting the use of nonstandard partially hydrolyzed formulas may not be appropriate for normal, healthy infants. A recent study demonstrated that feeding a partially hydrolyzed, low-lactose formula resolved symptoms in an infant population identified by fussy behavior [20]. Recommendations for the use of partially hydrolyzed formulas should be supported by clinical studies within the intended infant population. Further investigation and confirmation of partially hydrolyzed formula's effect on a fussy infant population is warranted.

## Conclusion

This study demonstrated no difference in tolerance of standard intact cow milk protein formula compared with partially hydrolyzed cow milk protein formula for healthy, term infants over a 60-day feeding period. A significant difference between study physician- and parent-assessed formula intolerance was observed, indicating parents discontinued formula for reasons other than formula intolerance. This conclusion parallels earlier reports that parents mistake behaviors common in early infancy (regurgitation, excessive crying, etc.) as manifestations of formula intolerance and unnecessarily switch formulas, often to non-standard formulas. Nonstandard formulas, such as partially hydrolyzed formulas, may be appropriate for a targeted group of individuals rather than as a first-choice formula best-suited for a healthy infant population.

## Abbreviations

DHA: docosahexaenoic acid; ARA: arachidonic acid; CMBF: cow milk-based formula; ICQ: Infant Characteristic Questionnaire; INTACT: intact cow milk protein formula; PH: partially hydrolyzed cow milk protein formula; CI: confidence interval

## Competing interests

CL Berseth, SH Mitmesser, JD Marunycz, and JA Vanderhoof are employees of the study sponsor, Mead Johnson Nutrition, Evansville, IN.

## Authors' contributions

CLB and JAV conceived of the study. CLB and EEZ participated in design and coordination of the study. JDM performed the statistical analyses. CLB, SHM, EEZ, JDM, and JAV helped draft the manuscript. All authors read and approved the final manuscript.
